# In vivo CRISPR Activation Screening, a Powerful Tool to Discover Oncogenic Driver Genes in Hepatocellular Carcinoma

**DOI:** 10.1016/j.jcmgh.2024.101459

**Published:** 2025-01-21

**Authors:** Shaimaa Gad, Ruisong Ye, Wei Qiu

**Affiliations:** Department of Surgery, Loyola University Chicago, Stritch School of Medicine, Maywood, Illinois; Department of Cancer Biology, Loyola University Chicago, Stritch School of Medicine, Maywood, Illinois; Department of Pharmacology, Medical Research and Clinical Studies Institute, National Research Center, Giza, Egypt; Department of Surgery, Loyola University Chicago, Stritch School of Medicine, Maywood, Illinois; Department of Cancer Biology, Loyola University Chicago, Stritch School of Medicine, Maywood, Illinois

Hepatocellular carcinoma (HCC) depicts a critical challenge worldwide as the most prevalent liver cancer and the deadliest liver disease.^1^ HCC heterogenous tumors demonstrate genetic and epigenetic alterations that require multi-disciplinary remedial methodology and restrict the effectiveness of the currently available treatments.^2^ Despite the development of some effective targeted therapies for HCC, the long-term survival benefit for advanced-stage disease remains limited.^3^ Therefore, it is imperative to identify molecular oncogenic drivers that can be targeted to improve the therapeutic outcomes for patients with HCC.

The recent study by Vázquez Salgado et al^4^ reveals promising findings for 3 potential vulnerable targets in the tumor promotor pathways incurred in advanced HCC, accounting for progress in HCC genetic mapping and helping the future development of effective therapeutic strategies for this challenging disease. The authors hypothesized that oncogenic drivers of HCC can be identified by the amplified genetic signature expressed in HCC tumors. Using the The Cancer Genome Atlas (TCGA) database, the authors recognized 51 genes that are frequently amplified in human HCC, all residing on human chromosome 1q and 8q. To assess their contribution to HCC tumorigenesis, in vivo CRISPR activation (CRISPRa) screens were utilized as a state-of-the-art technique to activate these targeted genes, and *MRPL9p, VPS72*, and *GBA1* were identified as drivers of liver tumorigenesis. The authors further employed single-gene CRISPRa mouse models to explore their unique function in HCC. Hyperactivation of any of these genes promoted *MYC*-driven tumorigenesis, hyperproliferation, and increased tumor burden and mortality rates. Moreover, the authors performed bulk RNA sequencing analysis to explore the underlying specific mechanisms of these genes.

Combined with the TCGA database and in vivo CRISPRa screening, this study systematically performed a clinically relevant, HCC-focused screening to identify the drivers of liver tumorigenesis. CRISPRa screening provides a powerful platform to identify and validate gene functions. Its ability to perform gain-of-function screens while maintaining the natural genetic context of genes makes it particularly suited for addressing questions such as identifying critical genes for liver tumorigenesis. Unlike CRISPR knockout screens, CRISPRa screens upregulate genes rather than disrupt them. This approach avoids the risk of compensatory mechanisms or lethality often observed in knockout studies, making it particularly useful for essential genes for cell survival. As with any CRISPR-based technology, off-target effects need to be considered for the CRISPRa screens. However, this study demonstrated limited off-targets for this state-of-the-art technology by validating the functions of these identified oncogenes in hepatocarcinogenesis. It would be expected that as technologies for in vivo screening and delivery advance, the utility of CRISPRa screens in liver cancer research will only expand, making them a cornerstone for cancer biology and therapy development.

This study discovered that *MRPL9, VSP72,* and *GBA1* promote *Myc*-driven HCC development. However, highlighting the role of *MRPL9* in HCC was the most innovative. The *MRPL9* gene encodes the mitochondrial ribosomal protein L9, a component of the 39S subunit of the mitochondrial ribosome. MRPL9 is essential for translating mitochondrial-encoded proteins, which are key components of the oxidative phosphorylation system.^5^ MRPL9 was recently suggested as a reliable circulating diagnostic biomarker and therapeutic target for HCC.^5^ However, the role of MPRL9 in liver tumorigenesis remained unknown. For the first time, this study convincingly demonstrated that MRPL9 activation significantly promotes Myc-driven HCC development, although the underlying molecular mechanisms remain unclear. The authors conducted bulk RNA sequencing and found that, of the top 100 dysregulated genes in MRPL9/MYC-hyperactivated tumors, 14% are correlated to mitochondrial function and ion transport, suggesting alteration of these signaling pathways could represent a mechanism of MRPL9/MYC-induced hepatocarcinogenesis. Of note, the top-ranked upregulated gene is *MYG1* (Melanocyte proliferating gene 1) and the most downregulated is *Echdc3* (enoyl-CoA hydratase domain containing 3), which showed similar expression trends in MRPL9+ human HCC tumors. In addition, cell cycle- and apoptosis-associated genes were also dysregulated, which might also explain the accelerated tumor growth and proliferation in MRPL9/MYC-hyperactivated tumors. Future studies are warranted to test these hypotheses.

This study also revealed that VSP72 and GBA1 promote c-Myc-driven HCC development. Although discovering the roles of these genes in Myc-driven HCC was compelling, the finding was not surprising. VPS72 (vacuolar protein sorting-associated protein 72 homolog) has been known to enable histone binding, chromatin remodeling, and cell cycle regulation.^6^ It has recently been identified as a potential target for HCC treatment and a prognosis biomarker.^6^^,^^7^ Specifically, Liu et al showed that overexpressing VPS72 promoted HCC initiation and progression by interacting with MYC and ACTL6A, forming the ACTL6A/MYC complex.^6^ Further, they found that VPS72/MYC-overexpressed tumors were enriched with genes involved in PIP2 regulation, phosphorus and lipid metabolism, and mitotic prometaphase, which could contribute to the acceleration of MYC-induced tumor growth by VPS72. GBA1 is a membrane protein involved in glycolipid metabolism, cleaving glucoceramides into ceramides essential for the endosomal-lysosomal system.^8^^,^^9^ Recently, GBA1 was identified as an oncogene and part of the autophagic degradation machinery in HCC.^10^ GBA1-mediated glucosylceramide reprogramming promotes liver cancer metastasis through the Wnt/β-catenin pathway.^11^ Further, *GBA1*/*MYC*-hyperactivated tumors revealed *Ocrl* as the most upregulated gene, which encodes a catalytic phosphatase for PIP2 hydrolysis^12^ and enables endosome trafficking,^13^ which could also represent a mechanism by which GBA1 promotes MYC-induced HCC development.

In summary, the study by Vázquez Salgado et al shed light on 3 amplified genes (*MRPL9p, VPS72*, and *GBA1)* in human HCC in promoting HCC tumorigenesis and hyperproliferation. The authors employed in vivo CRISPRa screens as a precise, sensitive, and innovative tool to identify the gain of function of these genes in HCC (Figure 1). The results suggested that *MRPL9p, VPS72*, and *GBA1* genes act as putative oncogenic co-drivers in HCC. However, their function as solo drivers is still to be demonstrated. Considering the shared chromosomal location and genetic signature, future studies on interaction and crosstalk between *VPS72*, *GBA1*, and *MRPL9* genes will be valuable for a better understanding their tumorigenic mechanisms in HCC. In addition, future targeting of *MRPL9p, VPS72*, and *GBA1,* as well as their signaling pathways, could aid drug discovery and development for the treatment of advanced HCC.

**Figure 1:**
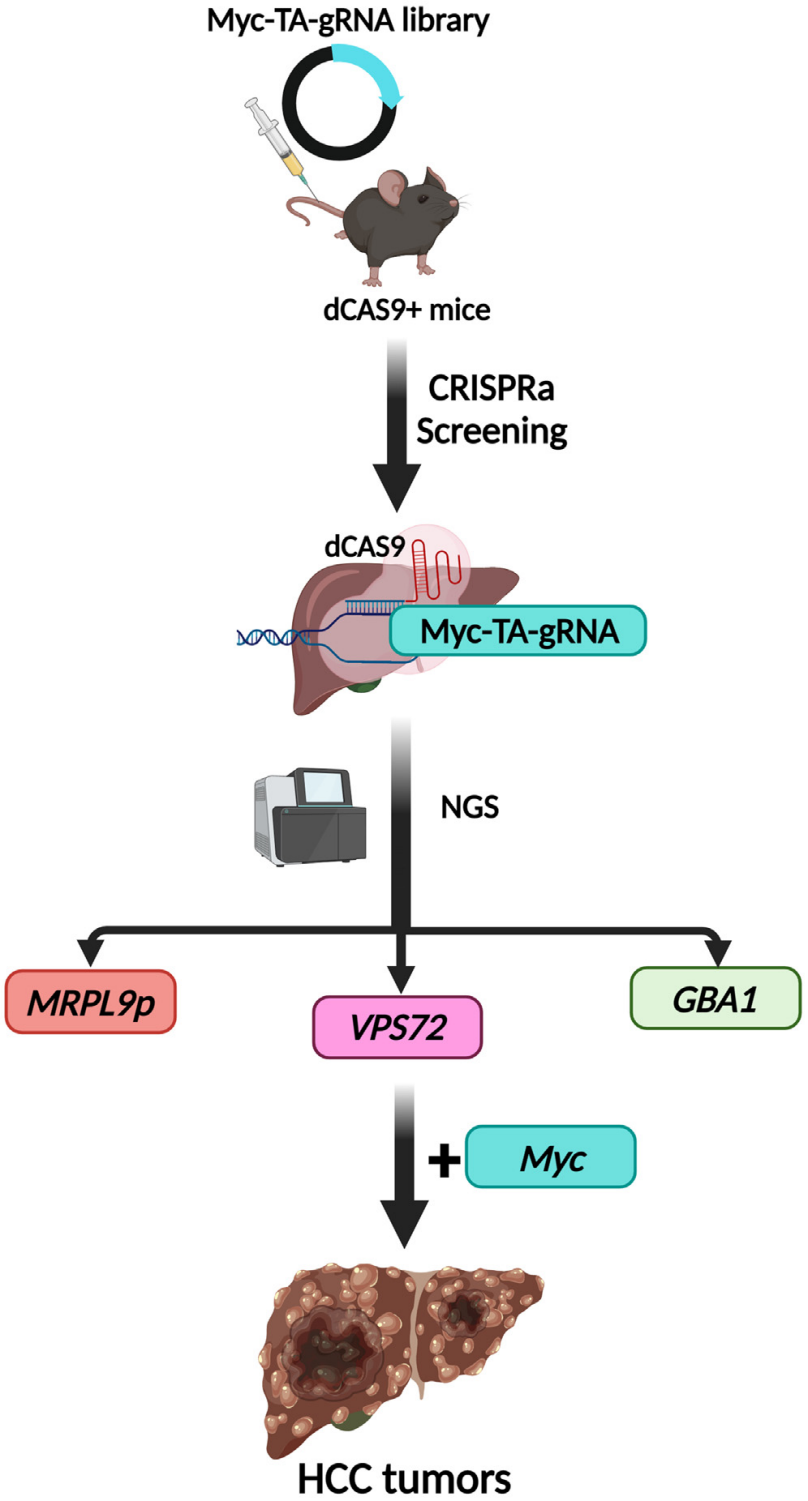
**Schematic representation of in vivo CRISPR activation (CRISPRa) screening platform reveals *MRPL9p, VPS72*, and *GBA1* as oncogenic drivers in HCC.** CRISPRa screen was conducted to directly annotate gene gain of function in HCC. Of 51 enriched genes, *MRPL9, VPS72*, and *GBA1* were identified to be hyperactivated in HCC tumors and promote Myc-driven HCC tumorigenesis. The illustration was created using BioRender.com.

## References

[1] Adlat S., Vazquez Salgado A.M., Lee M. (2023). Emerging and potential use of CRISPR in human liver disease. Hepatology.

[2] Dal Bo M., De Mattia E., Baboci L. (2020). New insights into the pharmacological, immunological, and CAR-T-cell approaches in the treatment of hepatocellular carcinoma. Drug Resist Updat.

[3] Mandlik D.S., Mandlik S.K., Choudhary H.B. (2023). Immunotherapy for hepatocellular carcinoma: current status and future perspectives. World J Gastroenterol.

[4] Vázquez Salgado AM, Cai C, Lee M (2025). In vivo CRISPR activation screening reveals chromosome 1q genes *VPS72*, *GBA1*, and *MRPL9* drive hepatocellular carcinoma. Cell Mol Gastroenterol Hepatol.

[5] Xie C., Hu J., Hu Q. (2023). Classification of the mitochondrial ribosomal protein-associated molecular subtypes and identified a serological diagnostic biomarker in hepatocellular carcinoma. Front Surg.

[6] Liu F., Liao Z., Qin L. (2023). Targeting VPS72 inhibits ACTL6A/Myc axis activity in HCC progression. Hepatology.

[7] Huang J., Gan J., Wang J. (2023). VPS72, a member of VPS protein family, can be used as a new prognostic marker for hepatocellular carcinoma. Immun Inflamm Dis.

[8] Magalhaes J., Gegg M.E., Migdalska-Richards A. (2016). Autophagic lysosome reformation dysfunction in glucocerebrosidase deficient cells: relevance to Parkinson disease. Hum Mol Genet.

[9] Trajkovic K., Hsu C., Chiantia S. (2008). Ceramide triggers budding of exosome vesicles into multivesicular endosomes. Science.

[10] Chen W., Ma Z., Yu L. (2022). Preclinical investigation of artesunate as a therapeutic agent for hepatocellular carcinoma via impairment of glucosylceramidase-mediated autophagic degradation. Exp Mol Med.

[11] Qiu Z., Wang X., Yang Z. (2022). GBA1-dependent membrane glucosylceramide reprogramming promotes liver cancer metastasis via activation of the Wnt/β-catenin signalling pathway. Cell Death Dis.

[12] Bohdanowicz M., Balkin D.M., De Camilli P., Grinstein S. (2012). Recruitment of OCRL and Inpp5B to phagosomes by Rab5 and APPL1 depletes phosphoinositides and attenuates Akt signaling. Mol Biol Cell.

[13] Vicinanza M., Di Campli A., Polishchuk E. (2011). OCRL controls trafficking through early endosomes via PtdIns4,5P(2)-dependent regulation of endosomal actin. EMBO J.

